# 6-(4-Methyl­phen­yl)-1,3,5-triazine-2,4-di­amine–benzoic acid (1/1)

**DOI:** 10.1107/S1600536813013883

**Published:** 2013-05-25

**Authors:** Kaliyaperumal Thanigaimani, Nuridayanti Che Khalib, Ibrahim Abdul Razak, Palanisamy Lavanya, Kasthuri Balasubramani

**Affiliations:** a School of Physics, Universiti Sains Malaysia, 11800 USM, Penang, Malaysia; b Department of Chemistry, Government Arts College (Autonomous), Thanthonimalai, Karur 639 005, Tamil Nadu, India

## Abstract

The benzoic acid mol­ecule of the title adduct, C_10_H_11_N_5_·C_7_H_6_O_2_, is approximately planar, with a dihedral angle of 7.2 (3)° between the carb­oxy­lic acid group and the benzene ring. In the triazine mol­ecule, the plane of the triazine ring makes a dihedral angle of 28.85 (9)° with that of the adjacent benzene ring. In the crystal, the two components are linked by N—H⋯O and O—H⋯N hydrogen bonds with an *R*_2_^2^(8) motif, thus generating a 1 + 1 unit of triazine and benzoic acid mol­ecules. The acid–base units are further connected by N—H⋯N hydrogen bonds with *R*_2_^2^(8) motifs, forming a supra­molecular ribbon along [101]. The crystal structure also features weak π–π [centroid–centroid distances = 3.7638 (12) and 3.6008 (12) Å] and C—H⋯π inter­actions.

## Related literature

For the biological activity of triazine derivatives, see: Bork *et al.* (2003 ▶). For related structures, see: Thanigaimani *et al.* (2007 ▶, 2012*a* ▶,*b* ▶). For hydrogen-bond motifs, see: Bernstein *et al.* (1995 ▶). For bond-length data, see: Allen *et al.* (1987 ▶). For stability of the temperature controller used for the data collection, see: Cosier & Glazer (1986 ▶).
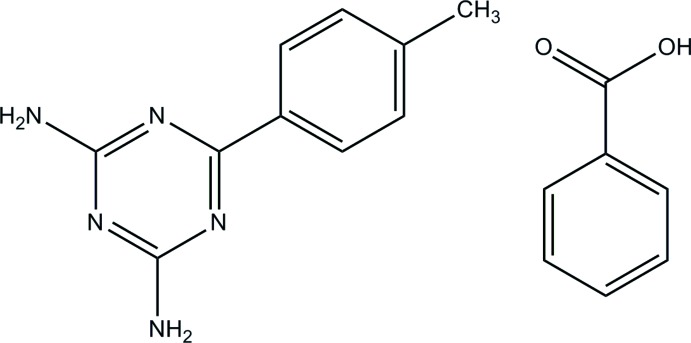


## Experimental

### 

#### Crystal data

C_10_H_11_N_5_·C_7_H_6_O_2_
*M* *_r_* = 323.36
Triclinic, 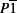

*a* = 7.4324 (5) Å
*b* = 10.9717 (3) Å
*c* = 11.2267 (3) Å
α = 117.202 (1)°
β = 101.645 (2)°
γ = 94.032 (2)°
*V* = 783.47 (6) Å^3^
*Z* = 2
Mo *K*α radiation
μ = 0.09 mm^−1^
*T* = 100 K
0.53 × 0.43 × 0.21 mm

#### Data collection

Bruker SMART APEXII CCD area-detector diffractometer
Absorption correction: multi-scan (*SADABS*; Bruker, 2009 ▶) *T*_min_ = 0.952, *T*_max_ = 0.980
16402 measured reflections
4578 independent reflections
3744 reflections with *I* > 2σ(*I*)
*R*_int_ = 0.028

#### Refinement

*R*[*F* ^2^ > 2σ(*F* ^2^)] = 0.062
*wR*(*F* ^2^) = 0.157
*S* = 1.11
4578 reflections
238 parameters
1 restraint
H atoms treated by a mixture of independent and constrained refinement
Δρ_max_ = 0.38 e Å^−3^
Δρ_min_ = −0.40 e Å^−3^

### 

Data collection: *APEX2* (Bruker, 2009 ▶); cell refinement: *SAINT* (Bruker, 2009 ▶); data reduction: *SAINT*; program(s) used to solve structure: *SHELXTL* (Sheldrick, 2008 ▶); program(s) used to refine structure: *SHELXTL*; molecular graphics: *SHELXTL*; software used to prepare material for publication: *SHELXTL* and *PLATON* (Spek, 2009 ▶).

## Supplementary Material

Crystal structure: contains datablock(s) global, I. DOI: 10.1107/S1600536813013883/is5271sup1.cif

Structure factors: contains datablock(s) I. DOI: 10.1107/S1600536813013883/is5271Isup2.hkl

Supplementary material file. DOI: 10.1107/S1600536813013883/is5271Isup3.cml

Additional supplementary materials:  crystallographic information; 3D view; checkCIF report

## Figures and Tables

**Table 1:** Hydrogen-bond geometry (Å, °) *Cg*2 is the centroid of the C2–C7 ring.

*D*—H⋯*A*	*D*—H	H⋯*A*	*D*⋯*A*	*D*—H⋯*A*
N4—H1*N*4⋯N2^i^	0.839 (19)	2.19 (2)	3.021 (2)	172 (2)
N4—H2*N*4⋯O2^ii^	0.86 (3)	2.11 (3)	2.965 (3)	172 (3)
N5—H1*N*5⋯N3^iii^	0.85 (3)	2.14 (3)	2.984 (3)	169 (3)
O1—H1*O*1⋯N1^iv^	0.83 (3)	1.80 (3)	2.613 (2)	167 (3)
C1—H1*B*⋯*Cg*2^v^	0.98	2.75	3.661 (2)	156

Symmetry codes: (i) 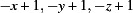; (ii) 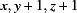; (iii) 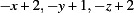; (iv) 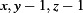; (v) 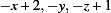.

**Fig. 1.**
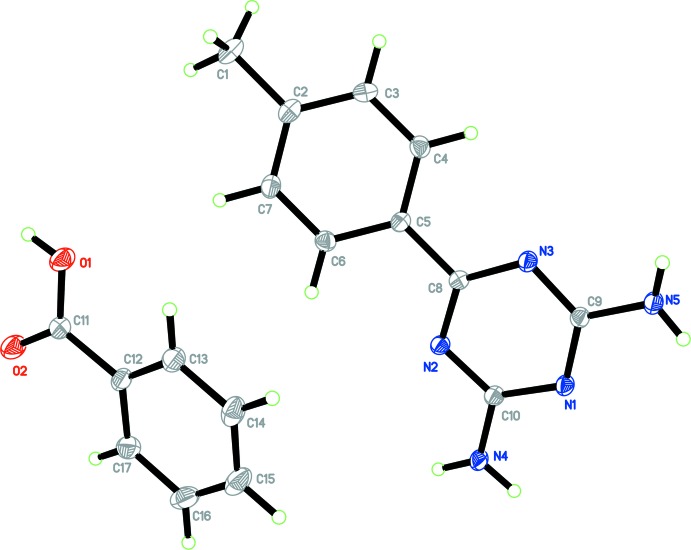
The molecular structure of the title compound with atom labels with 50% probability displacement ellipsoids.

**Fig. 2.**
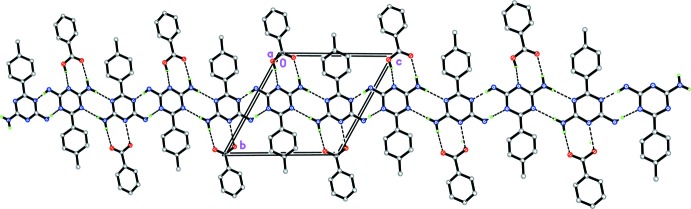
The crystal packing of the title compound. The H atoms not involved in the intermolecular interactions (dashed lines) have been omitted for clarity.

## supplementary crystallographic information

### Comment

Triazine derivatives show antitumor activity, as well as a broad range of biological activities, such as anti-angiogenesis and antimicrobial effects (Bork *et al.*, 2003). Related crystal structures of 2,4-diamino-6-phenyl-1,3,5-triazine- sorbic acid (1/1) (Thanigaimani *et al.*, 2007), 6-(4-methoxyphenyl)-1,3,5-triazine-2,4-diamine (Thanigaimani *et al.*, 2012*a*) and adipic acid-2.4-diamino- 6-(4-methoxyphenyl)-1,3,5-triazine (1/2) (Thanigaimani *et al.*, 2012*b*) have been reported. In the present study, hydrogen-bonding patterns in the 2,4-diamino-6-(4-methylphenyl)-1,3,5-triazine-benzoic acid (1/1) co-crystal are investigated.

The asymmetric unit (Fig. 1) contains one 2,4-diamino-6-(4-methylphenyl)-1,3,5-triazine molecule and one benzoic acid molecule. The dihedral angle between the triazine ring [N1/C10/N2/C8/N3/C9, maximum deviation = 0.006 (2) Å for atoms N2 & C10] and the plane formed by the benzoic acid molecule (O1/O2/C11–C17) is 11.16 (7)°. The triazine ring forms dihedral angle of 28.85 (9)° with the benzene ring (C2–C7). The bond lengths (Allen *et al.*, 1987) and angles are normal.

In the crystal (Fig. 2), the triazine molecules are base-paired [with a graph-set (Bernstein *et al.*, 1995) of *R*_2_^2^(8)] on either side *via* N4—H1N4···N2^i^ and N5—H1N5···N3^iii^ hydrogen bonds (symmetry codes in Table 1), forming a supramolecular ribbon. Each triazine molecule interacts with the carboxyl group of benzoic acid molecule *via* N4—H2N4···O2^ii^ and O1—H1O1···N1^iv^ hydrogen bonds (symmetry codes in Table 1), generating *R*_2_^2^(8) ring motifs. The crystal structure is further stabilized by π–π interactions between the benzene (C*g*2; C2–C7) rings [C*g*2···C*g*2= 3.7638 (12) Å; 1 - *x*, -*y*, 1 - *z*] and that between triazine (C*g*1; N1/C9/N3/C8/N2/C10) and benzene rings (C*g*3; C12–C17) [C*g*1···C*g*3= 3.6008 (12) Å; 2 - *x*,1 - *y*, 1 - *z*] and C—H···π interactions (Table 1) involving the C2–C7 (centroid *Cg*2) ring.

### Experimental

Hot methanol solutions (20 ml) of 2,4-diamino-6-(4-methylphenyl)-1,3,5-triazine (50 mg, Aldrich) and benzoic acid (31 mg, Aldrich) were mixed and warmed over a heating magnetic stirrer hotplate for a few minutes. The resulting solution was allowed to cool slowly at room temperature and crystals of the title compound (I) appeared after a few days.

### Refinement

O– and N-bound H atoms were located in a difference Fourier maps. Atoms H1N4, H2N4, H1N5 and H2N5 were refined freely, while atom H1O1 was refined with a bond restraint O—H = 0.82 (1) Å [refined distances: N4—H1N4 = 0.84 (2) Å, N4—H2N4 = 0.86 (3) Å, N5—H1N5 = 0.85 (3) Å, N5—H2N5 = 0.80 (3) Å and O1—H1O1 = 0.833 (10) Å]. The remaining hydrogen atoms were positioned geometrically (C—H = 0.95–0.98 Å) and were refined using a riding model, with *U*_iso_(H) = 1.2*U*_eq_(C) or 1.5*U*_eq_(methyl C). A rotating-group model was used for the methyl group. Six outliers (-4 3 0, -2 1 0, -3 -8 13, -2 6 0, -3 -3 12 and -6 4 0) were omitted in the final refinement.

### Crystal data

**Table:**

C_10_H_11_N_5_·C_7_H_6_O_2_	*Z* = 2
*M**_r_* = 323.36	*F*(000) = 340
Triclinic, *P*1	*D*_x_ = 1.371 Mg m^−^^3^
Hall symbol: -P 1	Mo *K*α radiation, λ = 0.71073 Å
*a* = 7.4324 (5) Å	Cell parameters from 7150 reflections
*b* = 10.9717 (3) Å	θ = 2.9–30.0°
*c* = 11.2267 (3) Å	µ = 0.09 mm^−^^1^
α = 117.202 (1)°	*T* = 100 K
β = 101.645 (2)°	Block, colourless
γ = 94.032 (2)°	0.53 × 0.43 × 0.21 mm
*V* = 783.47 (6) Å^3^	

### Data collection

**Table:**

Bruker SMART APEXII CCD area-detector diffractometer	4578 independent reflections
Radiation source: fine-focus sealed tube	3744 reflections with *I* > 2σ(*I*)
Graphite monochromator	*R*_int_ = 0.028
φ and ω scans	θ_max_ = 30.1°, θ_min_ = 2.1°
Absorption correction: multi-scan (*SADABS*; Bruker, 2009)	*h* = −10→10
*T*_min_ = 0.952, *T*_max_ = 0.980	*k* = −15→14
16402 measured reflections	*l* = −15→15

### Refinement

**Table:**

Refinement on *F*^2^	Primary atom site location: structure-invariant direct methods
Least-squares matrix: full	Secondary atom site location: difference Fourier map
*R*[*F*^2^ > 2σ(*F*^2^)] = 0.062	Hydrogen site location: inferred from neighbouring sites
*wR*(*F*^2^) = 0.157	H atoms treated by a mixture of independent and constrained refinement
*S* = 1.11	*w* = 1/[σ^2^(*F*_o_^2^) + (0.0412*P*)^2^ + 1.1661*P*] where *P* = (*F*_o_^2^ + 2*F*_c_^2^)/3
4578 reflections	(Δ/σ)_max_ < 0.001
238 parameters	Δρ_max_ = 0.38 e Å^−^^3^
1 restraint	Δρ_min_ = −0.40 e Å^−^^3^

### Special details

**Table:**

Experimental. The crystal was placed in the cold stream of an Oxford Cryosystems Cobra open-flow nitrogen cryostat (Cosier & Glazer, 1986) operating at 100.0 (1) K.
Geometry. All e.s.d.'s (except the e.s.d. in the dihedral angle between two l.s. planes) are estimated using the full covariance matrix. The cell e.s.d.'s are taken into account individually in the estimation of e.s.d.'s in distances, angles and torsion angles; correlations between e.s.d.'s in cell parameters are only used when they are defined by crystal symmetry. An approximate (isotropic) treatment of cell e.s.d.'s is used for estimating e.s.d.'s involving l.s. planes.
Refinement. Refinement of *F*^2^ against ALL reflections. The weighted *R*-factor *wR* and goodness of fit *S* are based on *F*^2^, conventional *R*-factors *R* are based on *F*, with *F* set to zero for negative *F*^2^. The threshold expression of *F*^2^ > σ(*F*^2^) is used only for calculating *R*-factors(gt) *etc*. and is not relevant to the choice of reflections for refinement. *R*-factors based on *F*^2^ are statistically about twice as large as those based on *F*, and *R*- factors based on ALL data will be even larger.

### Fractional atomic coordinates and isotropic or equivalent isotropic displacement parameters (Å^2^)

**Table:**

	*x*	*y*	*z*	*U*_iso_*/*U*_eq_	
N1	0.7322 (2)	0.67180 (16)	0.85847 (15)	0.0142 (3)	
N2	0.6434 (2)	0.46349 (15)	0.63917 (16)	0.0134 (3)	
N3	0.8105 (2)	0.45860 (16)	0.84190 (15)	0.0143 (3)	
N4	0.5792 (2)	0.67431 (17)	0.66117 (17)	0.0174 (3)	
N5	0.8918 (3)	0.66235 (18)	1.05089 (17)	0.0183 (3)	
O1	0.7991 (2)	−0.06107 (15)	0.03289 (15)	0.0225 (3)	
O2	0.6262 (2)	−0.02682 (15)	−0.13338 (15)	0.0238 (3)	
C1	0.7538 (3)	−0.1872 (2)	0.3826 (2)	0.0216 (4)	
H1A	0.7117	−0.2388	0.4268	0.032*	
H1B	0.8831	−0.1959	0.3788	0.032*	
H1C	0.6732	−0.2255	0.2881	0.032*	
C2	0.7436 (2)	−0.03535 (19)	0.4656 (2)	0.0161 (4)	
C3	0.7435 (3)	0.02206 (19)	0.6053 (2)	0.0165 (4)	
H3A	0.7479	−0.0357	0.6477	0.020*	
C4	0.7371 (3)	0.16236 (19)	0.68307 (19)	0.0154 (3)	
H4A	0.7377	0.1997	0.7780	0.018*	
C5	0.7297 (2)	0.24850 (18)	0.62243 (18)	0.0128 (3)	
C6	0.7296 (3)	0.19181 (19)	0.48277 (19)	0.0147 (3)	
H6A	0.7247	0.2495	0.4402	0.018*	
C7	0.7367 (3)	0.05171 (19)	0.40579 (19)	0.0169 (4)	
H7A	0.7369	0.0146	0.3110	0.020*	
C8	0.7265 (2)	0.39923 (18)	0.70582 (18)	0.0127 (3)	
C9	0.8102 (2)	0.59648 (18)	0.91395 (18)	0.0136 (3)	
C10	0.6516 (2)	0.60150 (18)	0.72027 (18)	0.0138 (3)	
C11	0.7236 (3)	0.0182 (2)	−0.01503 (19)	0.0173 (4)	
C12	0.7677 (3)	0.17012 (19)	0.0888 (2)	0.0174 (4)	
C13	0.8950 (3)	0.2206 (2)	0.2173 (2)	0.0192 (4)	
H13A	0.9552	0.1582	0.2411	0.023*	
C14	0.9343 (3)	0.3624 (2)	0.3110 (2)	0.0224 (4)	
H14A	1.0218	0.3970	0.3988	0.027*	
C15	0.8454 (3)	0.4537 (2)	0.2765 (2)	0.0258 (5)	
H15A	0.8724	0.5507	0.3409	0.031*	
C16	0.7172 (3)	0.4037 (2)	0.1480 (2)	0.0256 (5)	
H16A	0.6570	0.4663	0.1245	0.031*	
C17	0.6776 (3)	0.2617 (2)	0.0543 (2)	0.0211 (4)	
H17A	0.5893	0.2269	−0.0332	0.025*	
H1N4	0.527 (3)	0.638 (2)	0.576 (2)	0.012 (5)*	
H2N4	0.584 (4)	0.762 (3)	0.715 (3)	0.021 (6)*	
H1N5	0.965 (4)	0.621 (3)	1.082 (3)	0.026 (7)*	
H2N5	0.909 (4)	0.746 (3)	1.090 (3)	0.026 (7)*	
H1O1	0.762 (5)	−0.1439 (16)	−0.029 (3)	0.066 (11)*	

### Atomic displacement parameters (Å^2^)

**Table:**

	*U*^11^	*U*^22^	*U*^33^	*U*^12^	*U*^13^	*U*^23^
N1	0.0161 (7)	0.0122 (7)	0.0114 (7)	0.0041 (5)	0.0008 (5)	0.0042 (6)
N2	0.0143 (7)	0.0117 (7)	0.0125 (7)	0.0032 (5)	0.0009 (5)	0.0053 (6)
N3	0.0160 (7)	0.0125 (7)	0.0118 (7)	0.0039 (6)	0.0015 (5)	0.0045 (6)
N4	0.0241 (8)	0.0127 (7)	0.0109 (7)	0.0057 (6)	−0.0014 (6)	0.0041 (6)
N5	0.0250 (9)	0.0135 (7)	0.0120 (7)	0.0072 (6)	0.0001 (6)	0.0041 (6)
O1	0.0317 (8)	0.0136 (6)	0.0159 (7)	0.0043 (6)	−0.0003 (6)	0.0045 (5)
O2	0.0323 (8)	0.0166 (7)	0.0174 (7)	0.0055 (6)	0.0005 (6)	0.0063 (6)
C1	0.0202 (9)	0.0141 (8)	0.0263 (10)	0.0055 (7)	0.0070 (8)	0.0056 (8)
C2	0.0115 (8)	0.0136 (8)	0.0192 (9)	0.0025 (6)	0.0030 (6)	0.0050 (7)
C3	0.0159 (8)	0.0146 (8)	0.0206 (9)	0.0038 (7)	0.0043 (7)	0.0100 (7)
C4	0.0150 (8)	0.0163 (8)	0.0134 (8)	0.0029 (6)	0.0024 (6)	0.0066 (7)
C5	0.0112 (7)	0.0118 (7)	0.0132 (8)	0.0024 (6)	0.0016 (6)	0.0049 (6)
C6	0.0148 (8)	0.0159 (8)	0.0145 (8)	0.0042 (6)	0.0036 (6)	0.0081 (7)
C7	0.0185 (9)	0.0157 (8)	0.0137 (8)	0.0042 (7)	0.0046 (7)	0.0046 (7)
C8	0.0121 (8)	0.0118 (7)	0.0137 (8)	0.0022 (6)	0.0032 (6)	0.0059 (6)
C9	0.0145 (8)	0.0130 (8)	0.0123 (8)	0.0039 (6)	0.0032 (6)	0.0053 (6)
C10	0.0135 (8)	0.0132 (8)	0.0137 (8)	0.0033 (6)	0.0021 (6)	0.0061 (7)
C11	0.0201 (9)	0.0146 (8)	0.0167 (8)	0.0032 (7)	0.0058 (7)	0.0068 (7)
C12	0.0206 (9)	0.0125 (8)	0.0186 (9)	0.0032 (7)	0.0096 (7)	0.0053 (7)
C13	0.0211 (9)	0.0157 (9)	0.0200 (9)	0.0033 (7)	0.0078 (7)	0.0070 (7)
C14	0.0213 (9)	0.0176 (9)	0.0214 (9)	−0.0011 (7)	0.0085 (8)	0.0032 (8)
C15	0.0276 (11)	0.0136 (9)	0.0321 (11)	0.0003 (8)	0.0154 (9)	0.0050 (8)
C16	0.0294 (11)	0.0176 (9)	0.0370 (12)	0.0090 (8)	0.0179 (9)	0.0147 (9)
C17	0.0241 (10)	0.0190 (9)	0.0242 (10)	0.0059 (7)	0.0108 (8)	0.0117 (8)

### Geometric parameters (Å, º)

**Table:**

N1—C9	1.341 (2)	C3—C4	1.390 (3)
N1—C10	1.351 (2)	C3—H3A	0.9500
N2—C8	1.340 (2)	C4—C5	1.393 (3)
N2—C10	1.353 (2)	C4—H4A	0.9500
N3—C8	1.340 (2)	C5—C6	1.398 (2)
N3—C9	1.351 (2)	C5—C8	1.487 (2)
N4—C10	1.330 (2)	C6—C7	1.388 (3)
N4—H1N4	0.84 (2)	C6—H6A	0.9500
N4—H2N4	0.86 (3)	C7—H7A	0.9500
N5—C9	1.342 (2)	C11—C12	1.495 (3)
N5—H1N5	0.85 (3)	C12—C13	1.389 (3)
N5—H2N5	0.80 (3)	C12—C17	1.398 (3)
O1—C11	1.318 (2)	C13—C14	1.388 (3)
O1—H1O1	0.833 (10)	C13—H13A	0.9500
O2—C11	1.222 (2)	C14—C15	1.392 (3)
C1—C2	1.509 (3)	C14—H14A	0.9500
C1—H1A	0.9800	C15—C16	1.392 (3)
C1—H1B	0.9800	C15—H15A	0.9500
C1—H1C	0.9800	C16—C17	1.391 (3)
C2—C7	1.395 (3)	C16—H16A	0.9500
C2—C3	1.397 (3)	C17—H17A	0.9500
			
C9—N1—C10	115.80 (15)	C6—C7—H7A	119.5
C8—N2—C10	114.74 (15)	C2—C7—H7A	119.5
C8—N3—C9	114.64 (15)	N2—C8—N3	126.06 (16)
C10—N4—H1N4	122.6 (16)	N2—C8—C5	117.84 (15)
C10—N4—H2N4	116.8 (17)	N3—C8—C5	116.10 (15)
H1N4—N4—H2N4	121 (2)	N1—C9—N5	117.70 (16)
C9—N5—H1N5	117.4 (18)	N1—C9—N3	124.60 (16)
C9—N5—H2N5	117.2 (19)	N5—C9—N3	117.69 (17)
H1N5—N5—H2N5	119 (3)	N4—C10—N1	117.28 (16)
C11—O1—H1O1	108 (3)	N4—C10—N2	118.57 (16)
C2—C1—H1A	109.5	N1—C10—N2	124.15 (16)
C2—C1—H1B	109.5	O2—C11—O1	123.70 (18)
H1A—C1—H1B	109.5	O2—C11—C12	122.39 (18)
C2—C1—H1C	109.5	O1—C11—C12	113.91 (17)
H1A—C1—H1C	109.5	C13—C12—C17	120.07 (18)
H1B—C1—H1C	109.5	C13—C12—C11	121.28 (18)
C7—C2—C3	118.31 (17)	C17—C12—C11	118.65 (18)
C7—C2—C1	120.98 (18)	C14—C13—C12	119.9 (2)
C3—C2—C1	120.70 (18)	C14—C13—H13A	120.0
C4—C3—C2	121.04 (18)	C12—C13—H13A	120.0
C4—C3—H3A	119.5	C13—C14—C15	120.1 (2)
C2—C3—H3A	119.5	C13—C14—H14A	120.0
C3—C4—C5	120.29 (17)	C15—C14—H14A	120.0
C3—C4—H4A	119.9	C14—C15—C16	120.27 (19)
C5—C4—H4A	119.9	C14—C15—H15A	119.9
C4—C5—C6	119.00 (16)	C16—C15—H15A	119.9
C4—C5—C8	120.53 (16)	C17—C16—C15	119.7 (2)
C6—C5—C8	120.46 (16)	C17—C16—H16A	120.2
C7—C6—C5	120.42 (17)	C15—C16—H16A	120.2
C7—C6—H6A	119.8	C16—C17—C12	120.0 (2)
C5—C6—H6A	119.8	C16—C17—H17A	120.0
C6—C7—C2	120.94 (17)	C12—C17—H17A	120.0
			
C7—C2—C3—C4	−0.1 (3)	C10—N1—C9—N3	−0.8 (3)
C1—C2—C3—C4	179.00 (17)	C8—N3—C9—N1	0.7 (3)
C2—C3—C4—C5	0.3 (3)	C8—N3—C9—N5	179.86 (17)
C3—C4—C5—C6	−0.2 (3)	C9—N1—C10—N4	−177.53 (17)
C3—C4—C5—C8	−178.82 (17)	C9—N1—C10—N2	1.3 (3)
C4—C5—C6—C7	0.0 (3)	C8—N2—C10—N4	177.28 (17)
C8—C5—C6—C7	178.61 (17)	C8—N2—C10—N1	−1.5 (3)
C5—C6—C7—C2	0.1 (3)	O2—C11—C12—C13	−172.78 (19)
C3—C2—C7—C6	−0.1 (3)	O1—C11—C12—C13	7.2 (3)
C1—C2—C7—C6	−179.21 (17)	O2—C11—C12—C17	7.3 (3)
C10—N2—C8—N3	1.4 (3)	O1—C11—C12—C17	−172.72 (18)
C10—N2—C8—C5	−177.62 (16)	C17—C12—C13—C14	−0.7 (3)
C9—N3—C8—N2	−1.0 (3)	C11—C12—C13—C14	179.43 (18)
C9—N3—C8—C5	178.05 (16)	C12—C13—C14—C15	0.3 (3)
C4—C5—C8—N2	−152.42 (17)	C13—C14—C15—C16	−0.1 (3)
C6—C5—C8—N2	29.0 (2)	C14—C15—C16—C17	0.2 (3)
C4—C5—C8—N3	28.5 (2)	C15—C16—C17—C12	−0.6 (3)
C6—C5—C8—N3	−150.08 (17)	C13—C12—C17—C16	0.8 (3)
C10—N1—C9—N5	179.96 (17)	C11—C12—C17—C16	−179.30 (18)

### Hydrogen-bond geometry (Å, º)

*Cg*2 is the centroid of the C2–C7 ring.

**Table:**

*D*—H···*A*	*D*—H	H···*A*	*D*···*A*	*D*—H···*A*
N4—H1*N*4···N2^i^	0.839 (19)	2.19 (2)	3.021 (2)	172 (2)
N4—H2*N*4···O2^ii^	0.86 (3)	2.11 (3)	2.965 (3)	172 (3)
N5—H1*N*5···N3^iii^	0.85 (3)	2.14 (3)	2.984 (3)	169 (3)
O1—H1*O*1···N1^iv^	0.83 (3)	1.80 (3)	2.613 (2)	167 (3)
C1—H1*B*···*Cg*2^v^	0.98	2.75	3.661 (2)	156

Symmetry codes: (i) −*x*+1, −*y*+1, −*z*+1; (ii) *x*, *y*+1, *z*+1; (iii) −*x*+2, −*y*+1, −*z*+2; (iv) *x*, *y*−1, *z*−1; (v) −*x*+2, −*y*, −*z*+1.
